# Low Physical Activity Levels Are Linked to Early Hypertension Risk in College-Going Young Adults

**DOI:** 10.3390/healthcare9101258

**Published:** 2021-09-24

**Authors:** Kalyana Chakravarthy Bairapareddy, Mariam Mhd Salem Kamcheh, Ranim Jihad Itani, Mirna Mohamed, Heba Ayman Eid Abdellatif Zahran, Gopala Krishna Alaparthi, May Tamim, Parameshwar Anche, Baskaran Chandrashekaran

**Affiliations:** 1 Department of Physiotherapy, College of Health Sciences, University of Sharjah, Sharjah 27272, United Arab Emirates; u16107060@sharjah.ac.ae (M.M.S.K.); u16100637@sharjah.ac.ae (R.J.I.); u16104931@sharjah.ac.ae (M.M.); u15106311@sharjah.ac.ae (H.A.E.A.Z.); galaparthi@sharjah.ac.ae (G.K.A.); mtamim@sharjah.ac.ae (M.T.); 2 Department of Physiotherapy, Manipal College of Health Professions, Manipal Academy of Higher Education, Karnataka 576104, India; param.anche@gmail.com; 3 Department of Exercise Science & Sports, Manipal College of Health Professions, Manipal Academy of Higher Education, Karnataka 576104, India; baskaran.c@manipal.edu

**Keywords:** association, modifiable risk, physical activity, sedentary behaviour, young adults, blood pressure

## Abstract

**Background:** Sedentary behaviour and physical inactivity along with body mass are identified as critical determinants of vascular health along with body mass in young adults. However, the relationship between potential physical health and anthropometric variables with high blood Eid pressure remain unexplored in young adults from the United Arab Emirates region. **Methodology:** We administered a cross-sectional study in young adults assessing their self-reported physical activity levels, anthropometric variables (body mass index and waist circumference) and ambulatory blood pressure. The associations among potential physical health, anthropometric variables and high blood pressure were analysed through logistic regression after necessary transformation. **Results:** Of 354 participants (176 males, 178 females), we found 17.79% (*n* = 63) had higher mean arterial pressure. Males (*n* = 40; 22.73%) had higher risk of hypertension than females (*n* = 12.92%). Weekly physical activity levels (β = −0.001; *p* = 0.002), age (β = −0.168; *p* = 0.005) and gender (β = −0.709; *p* = 0.028) were found to be more strongly associated with hypertension risk than the body mass index (β = 0.093; *p* = 0.075), waist circumference (β = 0.013; *p* = 0.588) and the weekly sitting time (β = 0.000; *p* = 0.319) of the individuals. **Conclusions:** Lower physical activity was associated with hypertension risk compared to other modifiable risk factors such as waist circumference, body mass index and sedentary time in college-going young adults. Public health measures should continue to emphasise optimisation of weekly physical activity levels to mitigate vascular health risks at educational institution levels.

## 1. Introduction

High blood pressure is a predominant risk factor for chronic heart disease, stroke and coronary heart diseases that in turn lead to early mortality. The global average prevalence of higher blood pressure is 24.1% in men and 20.1% in women with increasing trends in Asia Pacific and low–middle-income countries [1]. Adults doubled their hypertension risk between 1975 (594 million) and 2015 (1.13 billion).

Epidemiological evidence has identified that non-modifiable risk factors (age, gender) and modifiable risk factors (body mass index, physical activity) play a crucial role in the pathogenesis of higher blood pressure [2,3]. As age increases, the hypertension risk doubles due to poor endothelial integrity and shear stress, calcification and autonomic disturbances [3]. Nevertheless, higher blood pressure risk is likely associated with male gender due to associated lifestyle factors such as diet, smoking and personality traits [4]. Further, high blood pressure risk was linked with high body mass index (BMI) and waist circumference (WC) [5]. Apart from the anthropometric variables, dietary intake, including low magnesium or potassium intake, was also found to influence the hypertension risk [6,7,8,9]. Elevated cholesterol, triglycerides that are associated with high BMI and WC, are likely to increase proatherogenesis and vascular dysfunction in later life.

For the past three decades, physical activity, which can be defined as meeting the national recommendations (weekly activity of 150 min of moderate to vigorous intensity exercise), was considered to be the key determinant for cardiovascular health [10,11,12,13]. Considerable evidence has substantiated that sedentary behaviour, defined as any waking behaviour (including lying or sitting) with low energy expenditure, likely increases the risk of hypertension [14,15]. Early experimental studies explored the independent role of prolonged sitting bouts in the pathogenesis of hypertension [16,17].

High blood pressure at adulthood (18–35 years) increases the risk of cardiovascular diseases and cognitive disorders such as Alzheimer’s and dementia [18,19]. Identification of the determinants may help to design interventions and ameliorate the morbidities and mortality associated with hypertension in later life. In spite of accumulating evidence on the high prevalence and the determinants of hypertension, the risk factors leading to hypertension in young adults of UAE is yet to be explored.

The aims of the present study were: (1) to determine the prevalence of hypertension and (2) explore whether both modifiable (physical activity, sitting time, waist circumference and body mass index) and non-modifiable (age, gender) risk factors contribute to the risk of hypertension in young adults in UAE.

## 2. Methods

### 2.1. Design

We administered a cross-sectional study in young adults of a multifaceted university aged 17–25 years who were recruited through notice boards and exchange user mail of the university. The ethical committee of the university (office of vice chancellor for research and graduate studies research ethics committee (REC-20-01-31-01-S)) approved the study. Necessary informed consent was obtained from the participants after explaining the study. The study conformed to the ethical principles as outlined in the Declaration of Helsinki. Young adults of both genders were included. The participants were excluded if they had a history of diabetes, hyperlipidemia, congenital heart diseases, collagen vascular diseases that are known to affect vascular health or any physical disability that might limit adequate participation in physical activity. Further, participants who could not speak or understand Arabic and English were excluded from study. On the assessment day, the participants reported to the exercise therapy lab of the university (maintained at 28*C and 60% humidity) from 7:00–8:00 a.m. before breakfast. Instructions, such as refraining from strenuous physical activity 24 h prior to test day, avoiding caffeinated drinks, smoking and adequate sleep for at least 8 h, were given the day prior to the test. The participants completed physical activity questionnaires, ambulatory blood pressure measurement and gave covariates such as socioeconomic status and anthropometric variables.

### 2.2. Anthropometry

Anthropometric measurements were taken (height to the nearest 0.1 cm and weight to the nearest 0.1 kg) using a stadiometer and weighing scales (Seca Middle East, Dubai, UAE). Height was measured in a standing position, with the head in the horizontal plane and heels together. Height measurements were repeated twice; if the difference between the first two was more than 0.5 cm, a third reading was taken. Weight was measured using an electronic weighing scale. Two weight measurements were obtained; if there was any difference in the first two readings by more than 0.3 kg, a third measurement was taken. Body mass index was calculated as kilograms per metres squared. Waist circumference was measured in the horizontal plane midway between the lowest rib and iliac crest using an inch tape (Seca 201, Seca Middle East, Dubai, UAE).

### 2.3. Physical Activity

Physical activity and sedentary behaviour (sitting time) were measured by the International Physical Activity Questionnaire—Short Form (IPAQ-SF). IPAQ-SF comprises seven questions with each question signaling the intensity, frequency and duration of three types of activity (light, moderate and vigorous) for activities of at least 10 min in the past seven days. Even if the participant did not exercise in the past 7 days, they responded with their sitting time to the question “During the last seven days, how much time did you spend sitting on a week day?” The scores were then transformed to metabolic equivalent (MET) minutes/week and analysed further.

### 2.4. Blood Pressure

Brachial artery blood pressure was measured using an oscillometric sphygmomanometer (Omron HEM 7124 CP, Omron Healthcare, Illinois, USA). The blood pressure measurements were administered as per the recommendations of the American Heart Association [20] and Lancet Commission on Hypertension group [21]. Participants were instructed to remain in the seated position for five minutes before measuring the BP to establish a steady state. The participants were asked to sit with their feet resting flat on a surface and their right arm resting at heart level. Based on arm circumference, the appropriate cuff was selected and placed around the upper arm. Three blood pressure measurements were taken with an interval of two minutes between two measurements. The mean systolic and diastolic blood pressure was calculated from the average of three blood pressure measurements. Subjects were classified as normotensive (<120/80), pre-hypertensive (120–139/80–189) or hypertensive (140\90 and higher) as per their BP level according to the World Health Organization (WHO) guidelines.

### 2.5. Variable Transformation

BMI was coded as “1” if less than 24.99 and “2” if >25. Gender was coded as “1” for males and “2” for females. Mean arterial pressure (MAP) was calculated using the formula: mean arterial pressure (MAP) = diastolic pressure + (1/3 × pulse pressure) where pulse pressure (mmHg) is the difference between systolic and diastolic blood pressures. Mean arterial pressure was coded as “0” (normal) if < 99.0 mmHg and “1” (high risk for hypertension) if >99.1 mmHg [22]. We have defined “risk of hypertension” or “hypertension risk” as any individual having a mean arterial pressure of >99.1 mmHg [22].

#### Statistical Analysis

The continuous variables (age, weight, body mass index, waist circumference, systolic, diastolic and mean blood pressures, self-reported sitting time and physical activity levels) and categorical variables (gender, body mass index, hypertension risk) were assessed for normality using a Shapiro–Wilk test. As normality was not found, we used a Mann–Whitney U test to compare the baseline characteristics across males and females.

We performed a logistic regression analysis to evaluate the association between the modifiable and non-modifiable risk factors and the hypertension risk. The continuous variables (waist circumference, body mass index, sitting time and physical activity levels as covariates) and their association with high blood pressure were assessed using logistic regression analysis and their odds ratios were explored. All the statistical analyses were carried out using statistical software (JASP v. 0.14.1, University of Amsterdam).

## 3. Results

We obtained 354 valid responses, constituting males (*n* = 176) and females (*n* = 178). The baseline characteristics of the respondents are reported as follows:

### 3.1. Baseline Characteristics

The mean age of the participants was 20.00 ± 1.83 years. Of them, 38.98% were found to be overweight or obese (*n* = 138) with a mean BMI of 24.14 ± 4.55. The mean waist circumference was found to be 80.87 ± 12.50 cm. The mean sitting time and physical activity in a week was estimated to be 1839.72 ± 791.78 min/week and 2080.97 ± 985.91 MET min/week, respectively. The average systolic blood pressure, diastolic pressure and mean arterial pressure were found to be 119.33 ± 11.92 mmHg, 76.52 ± 8.75 mmHg and 90.79 ± 9.13, respectively. We found that the prevalence of obesity in males (*n* = 80; 45.46%) was higher than females (*n* = 58; 32.58%). Of the participants, 17.79% (*n* = 63) had higher mean arterial blood pressure than the predicted normal level. We found males to be at higher risk for hypertension (*n* = 40; 22.73%) than females (*n* = 23; 12.92%). The baseline characteristics differed significantly between the genders, which is depicted in Table 1.

### 3.2. Determinants of Hypertension Risk (MAP > 99.1 mmHg)

**Figure 1:**
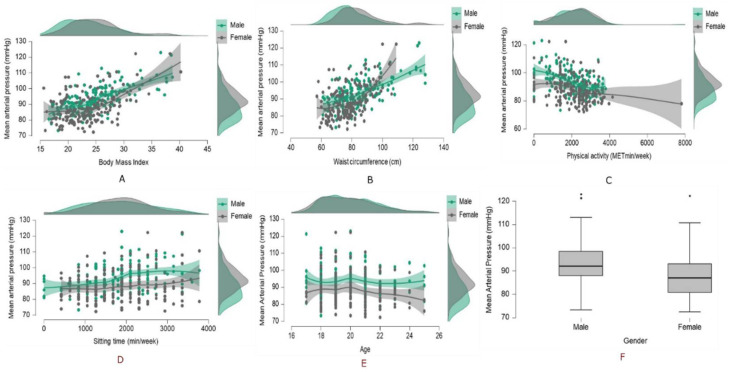
Modifiable risk factors, (**A**) body mass index, (**B**) waist circumference, (**C**) physical activity, (**D**) sitting time, (**E**) age, (**F**) gender, and their relation with the hypertension risk.

We included the determinants (gender, waist circumference, body mass index, weekly sitting time, age and physical activity) in a logistic regression model with mean arterial pressure as the dependent variable. We found a consistent and significant association of age, gender, BMI, waist circumference and physical activity levels with the hypertension risk, except the sitting time. The daily sitting time was not associated with hypertension when adjusted for age, gender, BMI or physical activity (Table 2). Figure 1 shows the association between the anthropometrics, activity levels and the early hypertension risk.

## 4. Discussion

Our cross-sectional study aimed to explore the prevalence of high blood pressure and its determinants in the young adults of UAE. We found that a significant proportion of the young adults of UAE had a high risk for hypertension (*n* = 63; 18%) with higher risk in males (*n* = 40; 22.73%) and lower risk in females (*n* = 23; 12.92%). Our study has established that higher blood pressure risk is associated with BMI, waist circumference, age, male gender and physical activity or could be predicted better by them than the daily sitting time.

### 4.1. Prevalence of Hypertension Risk in UAE Adults

We found that the prevalence of hypertension is 18% in young adults of UAE. Our results are marginally higher than the earlier epidemiological studies among young adults of Middle Eastern communities [23,24]. In a recent cross sectional study of 49 male and 71 female students at Jordan’s al Albayt University, risk of hypertension was shown to be prevalent in 16.33 percent of males and 11.27 percent of females [24]. While this study’s prevalence of high blood pressure was less than ours, it certainly shows that the risk of hypertension increased in males. A decade-old Saudi Arabian cross-sectional study revealed similar high blood pressure trends (*n* = 50; 13.5%) in Dammam College-going females [23].

### 4.2. Determinants of Hypertension Risk

Our study results showed that BMI, WC, age, male gender and physical activity are independent predictors for high blood pressure risk (map > 99.1 mmHg) but not the daily sitting time. Though age-related vascular changes, such as autonomic changes, endothelial integrity and baroreceptor control have been proposed, we have included only young adults with a small age difference. However, we could establish age as a better determinant for hypertension control, probably due to changes in healthy lifestyle factors such as weight change, diet, physical activity, alcohol and smoking which lead to atherogenesis and endothelial dysfunction [25]. Further, long working hours, lifestyle factors such as diet, smoking and alcohol and insomnia are more likely to increase the risk of high blood pressure in males than females [26]. Nevertheless, physical inactivity is likely to be an independent determinant for high blood pressure risk in young adults. Laboratory and epidemiological studies have found that acute or chronic physical activity facilitates post-exercise hypotension, baroreceptor sensitivity, renin–angiotensin regulation, endothelial nitric oxide production and long-term cardiac remodeling [27,28].

We found that males are more prone to early hypertension risk (MAP > 99.1 mmHg) than females, likely due to their large weight range and waist–hip circumference compared to the females, which was evident in our study. The increased weight or waist–hip circumference due to adiposity and consequent endothelial dysfunction due to renin–angiotensin–aldosterone dysregulation, poor shear stress and metabolic flexibility might be the putative mechanisms of the higher MAP in these young adults. Further, poor magnesium or potassium body reserves are also identified to be contributing factors which bridge the gap in the association between the high MAP and higher BMI or waist circumference in younger adults [29]. Nevertheless, it has long been recognised that sedentary behaviour is strongly associated with an increased risk of high blood pressure independent of physical activity but the potential mechanisms mediating this relation are less explored [25]. In our study, though daily sitting time is observed to increase blood pressure risk, we could not substantiate the relation statistically. We could probably explain this non-significance as the narrow age margin and high physical activity of the participants included in the study. We recommend future longitudinal trials to investigate the association between sedentary behaviour and the blood pressure risk in a larger UAE adult sample.

## 5. Limitations

A few limitations are worth mentioning: (1) we have not considered other risk factors such as sleep, socioeconomic status, education, smoking, alcohol intake, diet and mental health variables that could have an additive effect on the existing association between physical health and anthropometric variables with the hypertension risk [26]; (2) we have not measured physical activity or the sedentary behaviour objectively with accelerometers which might have facilitated a recall bias and in turn might have influenced the results; (3) our convenient sample with a narrow age range might have resulted in lower significance of the included variables for predicting the future hypertension risk.

## 6. Conclusions

We found a high prevalence of hypertension risk in college-going young adults. The potential determinants of high blood pressure risk were identified as age, gender, waist circumference, body mass index and physical activity, while sedentary time had a non-significant association with the hypertension risk. Educational institutions should advocate appropriate public health measures and policies mandating physical activity at the institutional level to mitigate the high blood pressure risk among college-going young adults.

## Figures and Tables

**Table 1:** Participant characteristics.

Baseline Variables	Males (*n* = 176)		Females (*n* = 178)		Significance
	Mean ± SD	Range		Range	
Age (years)	19.93 ± 1.887	17–25	20.05 ± 1.78	17–25	0.452
Weight (kgs)	75.46 ± 17.46	40.00–112.00	61.33 ± 12.82	46.00–96.00	<0.001
Body mass index (kg/m^2^)	25.00 ± 4.59	16.50–38.73	23.31 ± 4.36	15.60–40.20	<0.001
Waist circumference (cm)	84.58 ± 13.30	61–128	77.21 ± 10.47	57–109	<0.001
Physical activity time (MET min/week)	2107.33 ± 926.33	0–3800	2054.91 ± 1043.44	0–7812	0.399
Sitting time (min/week)	1833.93 ± 758.72	0–3780	1845.45 ± 825.27	420–3780	0.832
Systolic BP (mmHg)	123.18 ± 10.45	100–153	115.34 ± 12.09	93–150	<0.001
Diastolic BP (mmHg)	78.61 ± 7.75	59–108	74.45 ± 9.20	57–115	<0.001
Mean BP (mmHg)	93.46 ± 8.05	73.33–123.00	88.14 ± 9.38	72.33–122.33	<0.001

Note: All values are expressed as mean ± standard deviation; significance derived from Mann–Whitney U test. Abbreviations: BP = blood pressure.

**Table 2:** Logistic regression analysis of the potential determinants for the prediction of hypertension risk (MAP > 99.1).

Variable	Regression Coefficient	Odds Ratio (95% CI)
Gender	−0.709	0.492 (0.262–0.927) *
Age (years)	−0.168	0.845 (0.750–0.951) *
BMI	0.093	1.097 (0.947–1.272) **
WC	0.013	1.013 (0.966–1.063) *
ST (h/day)	0.000	1.000 (1.000–1.001)
PA (min/day)	−0.180	0.999 (0.999–1.000) **

BMI—Body mass index; WC—Waist circumference; ST—Sitting time; PA—Physical activity; * *p* < 0.01; ** *p* < 0.001.

## Data Availability

The data presented in this study are available on request from the corresponding author. Due to ethical concerns, the data is not publicly available.
